# Estimation on risk of spontaneous abortions by genomic disorders from a meta‐analysis of microarray results on large case series of pregnancy losses

**DOI:** 10.1002/mgg3.2181

**Published:** 2023-04-04

**Authors:** Gang Peng, Qinghua Zhou, Hongyan Chai, Jiadi Wen, Hongyu Zhao, Hugh S. Taylor, Yong‐Hui Jiang, Peining Li

**Affiliations:** ^1^ Department of Genetics Yale University School of Medicine New Haven Connecticut 06520 USA; ^2^ Department of Medical & Molecular Genetics Indiana University School of Medicine Indianapolis Indiana 46202 USA; ^3^ Department of Biostatistics School of Public Health, Yale University New Haven Connecticut USA; ^4^ Biomedical Translational Research Institute, Jinan University Guangzhou China; ^5^ Department of Obstetrics, Gynecology and Reproductive Sciences Yale University School of Medicine New Haven Connecticut 06520 USA

**Keywords:** chromosome microarray analysis (CMA), genomic disorders, pathogenic copy number variants (pCNVs), pregnancy loss (PL), products of conception (POC), spontaneous abortion (SAB)

## Abstract

A meta‐analysis on seven large case series (>1000 cases) of chromosome microarray analysis (CMA) on products of conceptions (POC) evaluated the diagnostic yields of genomic disorders and syndromic pathogenic copy number variants (pCNVs) from a collection of 35,130 POC cases. CMA detected chromosomal abnormalities and pCNVs in approximately 50% and 2.5% of cases, respectively. The genomic disorders and syndromic pCNVs accounted for 31% of the detected pCNVs, and their incidences in POC ranged from 1/750 to 1/12,000. The newborn incidences of these genomic disorders and syndromic pCNVs were estimated in a range of 1/4000 to 1/50,000 live births from population genetic studies and diagnostic yields of a large case series of 32,587 pediatric patients. The risk of spontaneous abortion (SAB) for DiGeorge syndrome (DGS), Wolf–Hirschhorn syndrome (WHS), and William–Beuren syndrome (WBS) was 42%, 33%, and 21%, respectively. The estimated overall risk of SAB for major genomic disorders and syndromic pCNVs was approximately 38%, which was significantly lower than the 94% overall risk of SAB for chromosomal abnormalities. Further classification on levels of risk of SAB to high (>75%), intermediate (51%–75%), and low (26%–50%) for known chromosomal abnormalities, genomic disorders, and syndromic pCNVs could provide evidence‐based interpretation in prenatal diagnosis and genetic counseling.

## Graphical abstract

Current introduction of enhanced non‐invasive prenatal screening (NIPT) and chromosome microarray analysis (CMA) has improved the diagnostic yield of pathogenic copy number variants (pCNVs) in prenatal diagnosis. However, the risk of spontaneous abortions (SABs) by genomic disorders and other syndromic pCNVs remains unknown. This study provided an estimation on the risk of SAB for major genomic disorders and syndromic pCNVs, which could be useful for laboratory result interpretation and informative genetic counseling.

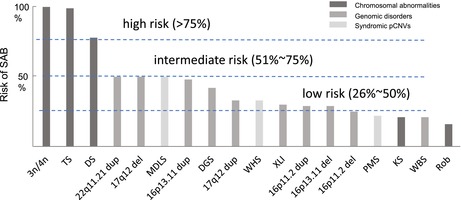

## 1 INTRODUCTION

The application of chromosome microarray analysis (CMA) by array comparative genomic hybridization (aCGH) or single‐nucleotide polymorphism (SNP) array has significantly improved the diagnostic accuracy and efficacy for chromosomal abnormalities and pathogenic copy number variants (pCNVs) in current prenatal and pediatric clinics (Chai et al., 2019; Wei et al., 2013). It is estimated that chromosomal abnormalities and pCNVs were detected in approximately 0.67% and 0.33% of newborns (Chai et al., 2019). CMA has also been introduced to detect chromosomal abnormalities and pCNVs in products of conceptions (POC) from pregnancy losses (Rajcan‐Separovic, 2012; Reddy et al., 2012;). Chromosomal abnormalities including mostly aneuploidies and polyploidies were detected in approximately 50% of POC (van den Berg et al., 2012; Zhou et al., 2016). A comparison of diagnostic findings from several meta‐analyses on small to large case series of CMA on POC revealed an additional yield of 2%–4% for pCNVs over karyotyping (Dhillon et al., 2014; Pauta et al., 2018;Martinez‐Portilla et al., 2019).

For conceptions with a chromosomal abnormality, it is well‐documented that 94% of them will end in a spontaneous abortion (SAB). The risk of SAB for triploidy/tetraploidy, monosomy X, trisomy 21, unbalanced rearrangements, other sex chromosome aneuploidies, and balanced rearrangement were 100%, 99%, 78%, 85%, 21%, and 16%, respectively (Nussbaum et al., 2016). However, the risk of SAB for pCNVs remains unknown due to significantly lower diagnostic yield of pCNVs in POC and lower newborn incidences of pCNVs than that of chromosomal abnormalities. The definition of pCNVs follows current technical standards and consensus recommendation of the American College of Medical Genetics and Genomics (ACMG) and the Clinical Genome Resource (ClinGen) (Riggs et al., 2020). In practice, pCNVs are classified as recurrent and nonrecurrent pCNVs while recurrent pCNVs include genomic disorders and other syndromic pCNVs (Girirajan et al., 2012; Yuan et al., 2021). Genomic disorders referred to microdeletion and microduplication syndromes and their recurrence is mediated by non‐allelic homologous recombination between locus‐specific low copy repeats. Syndromic pCNVs occurred mostly at the distal regions in the short arm or long arm of a chromosome and involved in one or a few critical regions for correlated syndromic phenotypes (Hao et al., 2022; Xie et al., 2022). Nonrecurrent pCNVs occurred sporadicallyin the genome with different gene content and variable phenotypes. This study is aimed to evaluate the diagnostic yields of genomic disorders and syndromic pCNVs in pregnancy loses through a systematic review of several large case series of CMA on POC and to estimate their risk of SAB through a comparison of their incidences between POC cases and pediatric patients. The results from this study could provide evidence‐based interpretation on the risk of SAB for prenatally detected genomic disorders and syndromic pCNVs and insight for potential genetic and pathologic etiology of pregnancy losses.

## 2 METHODS

Given the estimated 2% diagnostic yield of pCNVs in POC (Pauta et al., 2018), the literature review focused on large case series of CMA studies on more than 1000 cases of POC. The PubMed search used key terms CMA (aCGH and SNP array), POC, SAB, pregnancy losses, miscarriages, pCNVs, and genomic disorders for publications from January 2010 to May 2022. A total of nine studies met the criteria, and data were extracted from the main text and supplemental materials (Levy et al., 2014; Zhou et al., 2016; Sahoo et al., 2016; Chen et al., 2017; Maisenbacher et al., 2017; Peng et al., 2018; Li et al., 2020; Wang et al., 2021; Finley et al., 2022). Two studies from the same diagnostic service center with POC cases increased from 7396 to 24,900, and the data were extracted from the latter study (Finley et al., 2022; Sahoo et al., 2016). The study by Maisenbacher et al. (2017) on a large cohort of 22,451 POC samples focused on the incidence of the 22q11.2 deletion and lacked the data for other genomic disorders, which serves as an independent reference for the most common genomic disorder of DiGeorge syndrome (DGS, OMIM#188400). Common effect meta‐analysis was used to estimate the overall diagnostic yields of chromosomal abnormalities, pCNVs, and genomic disorders merging all seven studies (Barendregt et al., 2013). This study follows the MOOSE guideline, and a detailed MOOSE reporting checklist can be found in the Supplementary (Brooke et al., 2021).

The pCNVs detected from the seven large case series were re‐evaluated following the current ACMG/ClinGen technical standards and consensus recommendation (Riggs et al., 2020); pCNVs re‐classified as variants of uncertain significance or likely benign were excluded for further analysis. To make the results from POC comparable with findings from pediatric patients, pCNVs in 72 regions previously known to be associated with clinical phenotypes were selected (Girirajan et al., 2012; Yuan et al., 2021). These pCNVs include major recurrent genomic disorders and syndromic pCNVs. Other criteria for case inclusion were size cut‐off of less than 10 Mb, sole abnormality without other concurrent chromosomal rearrangements, and recurrence of three or more cases of the same pCNVs in one or more studies. To differentiate from chromosomal rearrangements detectable by karyotyping, the cut off size for pCNVs had been defined as <10 Mb (Levy et al., 2014). pCNVs concurrent with aneuploidies and large unbalanced rearrangements could be seen in up to 37% of cases (Finley et al., 2022). Since chromosomal abnormalities are known to cause SAB; the secondary pCNVs detected along with primary chromosomal abnormalities were excluded. Recurrence in three or more cases of the same pCNV from different studies or in one large case series is considered clinical evidence for an etiologic association with SAB, while pCNVs observed in only one and two cases were excluded to avoid bias in the calculation of the incidence and risk of SAB.

The incidences of genomic disorders and syndromic pCNVs in POC in each study were calculated using the detected case number of an individual pCNV divided by the total number of cases. The known newborn incidences of genomic disorders from population genetic studies were extracted from Online Mendelian Inheritance in Man (OMIM, https://www.omim.org/). The newborn incidences of other genomic disorders and syndromic pCNVs were calculated using the detected number of cases of an individual pCNV divided by an average newborn population estimated from the genomic disorders with known newborn incidences (Girirajan et al., 2012). The risks of SAB for genomic disorders and syndromic pCNVs were calculated by the number of cases in POC divided by the combined numbers of cases in POC and newborns based on a 15% of SAB and 85% of live births from 100,000 conceptions (Nussbaum et al., 2016). The difference between incidences in POC and newborns were compared by Fisher's exact test (*p* < 0.05).

## 3 RESULTS

### 3.1 Diagnostic yields of genomic disorders and syndromic pCNVs in POC

**FIGURE 1:**
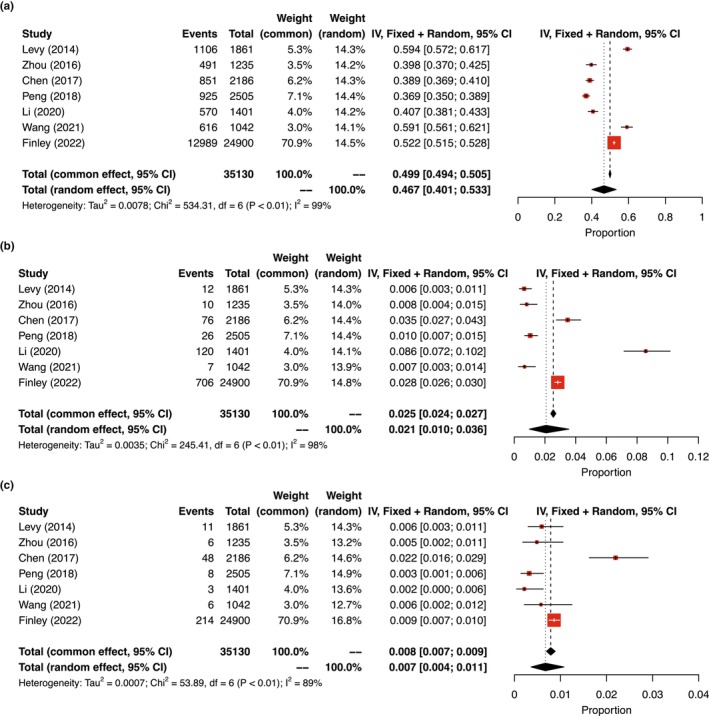
Summary of meta‐analysis results of the seven studies. Forest plots showed common and random effects for the overall diagnostic yields of (a) chromosomal abnormalities; (b) syndromic pCNVs; and (c) genomic disorders from POCs at 95% confidence interval (CI). In heterogeneity analysis, *τ* ^2^ is the estimate of the variance of the true effect sizes; *χ* ^2^ is the weighted sum of squared differences between individual study effects and the pooled effect across studies; and *I* ^2^ is the percentage of variation across studies that is due to heterogeneity rather than chance.

Of the seven studies, the cytogenomic findings from a total of 35,130 POC cases and results of meta‐analysis are summarized in Table 1 and Figure 1. The diagnostic yields for chromosomal abnormalities and pCNVs estimated from common effect meta‐analysis were approximately 49.9% (17,548 cases) and 2.5% (957 cases), respectively. Further classification of pCNVs showed a diagnostic yield of 0.8% (296 cases) for genomic disorders and syndromic pCNVs, which accounted for approximately 31% of the detected pCNVs. Lower yield of chromosomal abnormalities and higher yield of pCNVs noted in two studies were likely due to the classification of possibly large terminal and interstitial imbalances, derivative chromosomes, and complex rearrangements into pCNVs (Chen et al., 2017; Li et al., 2020).

**TABLE 1:** Diagnostic yields of chromosomal abnormalities and pCNVs from seven large case series of CMA on pregnancy losses.

Publications	Clinical specimens	Methods	Total cases	Chr Abns	(%)	pCNVs	(%)	GDs^a^	(%)
Levy et al. (2014)	Miscarriages (<20 weeks of gestation)	CMA (Illumina CytoSNP‐12)	1861	1106	59	12	0.64	11	0.59
Zhou et al. (2016)	POC from SAB	CMA (Agilent 60K aCGH)	1235	491	40	10	0.81	6	0.48
Chen et al. (2017)	POC from SAB	CMA (Version 7.6 Oligo, Baylor), WGS	2186	851	39	76	3.48	48	2.20
Peng and Yuan (2018)	POC from SAB	CMA (Affymetric CytoScan 750K)	2505	925	37	26	1.04	8	0.32
Li et al. (2020)	Miscarriages	CMA (Agilent aCGH), lc‐NGS	1401	570	41	120	8.57	3	0.14
Wang et al. (2021)	Miscarriages (<13 weeks of gestation)	CMA (Affymetric CytoScan 750K), QF‐PCR, HLPA	1042	616	59	7	0.67	6	0.60
Finley et al. (2022)	POC (fresh/FFPE) from SAB	CMA (Illumina CytoSNP‐12)	24,900	12,989	52	706	2.83	214	0.86
Total			35,130	17,548	49.90	957	2.50	296	0.80

Abbreviations: Chr Abns, chromosomal abnormalities; CMA, chromosome microarray analysis; HLPA, high‐throughput ligation‐dependent probe amplification; lcNGS, low‐coverage next‐generation sequencing; POC, products of conception; pCNVs, pathogenic copy number cvariants; SAB, spontaneous abortion; WGS, whole‐genome sequencing.

^a^ GDs, genomic disorders and syndromic pCNVs in Girirajan et al., 2012.

For the 296 cases with genomic disorders and syndromic pCNVs, the chromosomal regions, OMIM designation, genomic coordinates, and dosage sensitivity genes curated in ClinGen are listed in Table S1. Among them, 232 cases with three and more cases for 17 genomic disorders and four syndromic pCNVs met the inclusion criteria for further analysis, 43 cases with pCNVs in 10 loci occurring in three to seven cases were excluded due to the lack of matched data from the large pediatric case series (Girirajan et al., 2012), and 21 cases with pCNVs in 19 loci occurring once or twice were also excluded. The excluded cases included distal deletions of 9p, 11q (JS, Jacobsen syndrome, OMIM#147791), 14q, 15q, 18p and 18q as well as recurrent genomic disorders of a deletion at 15q11.2q13.1 (PWS/AS, Prader‐Willi syndrome/Angelman syndrome, OMIM#176270/105830), and 15q13.3 deletion syndrome (OMIM#612001). The newborn incidences of JS and PWS are 1/100,000 and 1/29,000, respectively (Ji et al., 2010; Whittington et al., 2001). The observation of a sole case in this collection of POC cases suggested an incidence of 1/35,000, which is higher than the newborn incidence of JS, but like that of PWS.

The 232 cases selected for further analysis included 17 recurrent genomic disorders and four syndromic pCNVs. These genomic disorders were at 1q21.1, 7q11.23, 15q11.2, 16p13.11,16p11.2, 17q12, 22q11.2, 22q11.2 distal, and Xp22.33. The four syndromic pCNVs were Wolf–Hirschhorn syndrome by a deletion at 4p16.3 (WHS, OMIM#194190), Miller–Dieker lissencephaly syndrome (MDLS, OMIM#247200) by a deletion at 17p13.3, Phelan–McDermid syndrome by a deletion of 22q13.3 (PMS, OMIM#606232), and a duplication of 22q13.3 (OMIM#615538).

### 3.2 Incidences of major genomic disorders and syndromic pCNVs in POC and livebirths

The incidences of the selected genomic disorders and syndromic pCNVs in POC, directly calculated using their case number divided by the total POC case number, are summarized in Table 2. The incidences ranged from 1/11,700 (3/35,130) for WHS to 1/750 (47/35,130) for 15q11.2 deletion syndrome. For genomic disorders, the incidences in POC for DGS, Williams–Beuren syndrome (WBS, OMIM#194050), and X‐linked ichthyosis (XLI, OMIM#308100) were 1/1000, 1/4400, and 1/2200 males, respectively. For syndromic pCNVs, the incidences in POC for WHS, MDLS, and PMS were 1/11,700, 1/5900, and 1/8800, respectively. Collectively, the overall incidence of these genomic disorders and syndromic pCNVs was 1/150.

**TABLE 2:** Incidences of major genomic disorders and syndromic pCNVs detected in POC and newborns.

Genomic disorders and syndromic pCNVs	OMIM	Cases in POC (*n*)	Incidence in POC (*n*/35,130)	Cases in pediatrics (*n*)	Incidence in newborns (*n*/724,000)	*p* value (<0.05)
Genomic disorders						
15q11.2 deletion syndrome (BP1‐BP2)	615656	47	1/750	166	1/4400	*
22q11.21 deletion syndrome (DiGeorge syndrome, DGS)	188400	35	1/1000	175	**1/4000**	*
22q11.21 duplication syndrome	608363	23	1/1500	87	1/8300	*
16p13.11 deletion		5	1/7000	45	1/16,100	0.08
16p13.11 duplication		24	1/1500	98	1/7400	*
16p11.2 deletion syndrome	611913	12	1/2900	125	1/5800	*
16p11.2 duplication syndrome	614671	10	1/3500	83	1/8700	*
1q21.1 deletion syndrome	612474	4	1/8800	100	1/7200	1
1q21.1 duplication syndrome	612475	10	1/3500	81	1/8900	*
7q11.23 deletion (Williams–Beuren syndrome, WBS)	194050	8	1/4400	83	**1/7500**	0.07
17q12 deletion syndrome	614527	7	1/5000	26	1/27,900	*
17q12 duplication syndrome	614526	4	1/8800	35	1/20,700	0.11
22q11.2 distal deletion syndrome	611867	4	1/8800	26	1/27,900	*
22q11.2 distal duplication		7	1/5000	18	1/40,200	*
16p12.1 deletion syndrome	136570	6	1/5900	56	1/12,900	0.06
Xp22.31 deletion (X‐linked Ichthyosis in male, XLI)	308100	6	1/2200 males	na	**1/6000 males**	nt
15q13.3 duplication		4	1/8800	27	1/26,800	0.05
Syndromic pCNVs						
4p16.3 deletion (Wolf–Hirschhorn syndrome, WHS)	194190	3	1/11,700	17	**1/50,000**	0.06
17p13.3 deletion syndrome (Miller–Dieker lissencephaly syndrome, MDLS)	247200	6	1/5900	21	1/34,500	*
22q13 deletion (Phelan–Mcdermid syndrome, PMS)	606232	4	1/8800	59	1/12,300	0.54
22q13 duplication syndrome	615538	3	1/11,700	3	1/241,300	*
Total		232	1/150	1331	1/540	*

*Note*: All calculated incidences round up to 100; newborn incidences in bold from population genetic studies.

Abbreviations: na, not available; nt, not test.

* *p* < 0.05.

The newborn incidences from population genetic studies for DGS, WBS, XLI, and WHS are 1/4000, 1/7500, 1/6000 males, and 1/50,000, respectively (Botto et al., 2003; Goodship et al., 1998; Maas et al., 2008; Shapiro et al., 1978; Stromme et al., 2002). A large case series of 32,587 pediatric patients from a 3‐year interval detected pCNVs in 2312 patients (Girirajan et al., 2012). Using the number of patients diagnosed with DGS, WBS, and WHS multiplied by their known newborn incidences, it was estimated that this large case series was from 700,000, 622,500, and 850,000 newborns, which gave an average of 724,000 newborns. The newborn incidences for other genomic disorders and syndromic pCNVs estimated using the number of diagnosed patients divided by the average newborns are summarized in Table 2. The newborn incidences of these genomic disorders and syndromic pCNVs were in the range of 1/4000 for DGS to 1/50,000 for WHS, with the only exception of 1/241,300 for 22q13 duplication. The newborn incidences of 10 genomic disorders and two syndromic pCNVs were significantly lower than that of POC (*p* < 0.05); other five genomic disorders and two syndromic pCNVs also had a lower incidence in newborn than in POC, but statistically insignificant. The overall newborn incidence of these genomic disorders and syndromic pCNVs was 1/540, which is three to four times less than their overall incidence of 1/150 in POC (*p* < 0.05).

### 3.3 Estimation on risk of SAB for major genomic disorders

It is generally accepted that approximately 15%–20% of clinically recognized pregnancies resulted in a miscarriage; this is considered a baseline risk of SAB for all pregnancies (Finley et al., 2022; Nussbaum et al., 2016). The higher incidences of major genomic disorders and syndromic pCNVs in POC than in newborns indicated an increased risk of SAB for fetuses carrying these genomic imbalances. The risk can be theoretically estimated from 100,000 conceptions by assuming a 15% of SAB (15,000) and 85% of live births (85,000). The risk of SAB for major genomic disorders and syndromic pCNVs, estimated as the number of cases in conceptions divided by the total count of cases in conceptions and live births, is shown in Table 3. The risk of SAB for these genomic disorders ranged from 21% for WBS to 60% for 22q11.2 distal duplication. The risk of SAB for syndromic pCNVs showed 21%, 33%, 50%, and 50% for PMS, WHS, MDLS, and 22q13 duplication, respectively. Given a 15%–20% risk for SAB for pregnancies, the risk of SAB of 21%–25% for WBS, PMS, and 16p11.2 deletion showed no increased risk. The overall risk of SAB for these genomic disorders and syndromic pCNVs was 38%.

**TABLE 3:** Outcome major genomic disorders and syndromic pCNVs of 100,000 conceptions.

Outcome	Conceptions	No. of SAB	Risk of SAB (%)	Live births
	100,000	15,000	15	85,000
Major genomic disorders				
15q11.2 deletion syndrome (BP1‐BP2)	39	20	51	19
22q11.21 deletion syndrome (DiGeorge syndrome, DGS)	36	15	42	21
22q11.21 duplication syndrome	20	10	50	10
16p13.11 deletion	7	2	29	5
16p13.11 duplication	21	10	48	11
16p11.2 deletion syndrome	20	5	25	15
16p11.2 duplication syndrome	14	4	29	10
1q21.1 deletion syndrome	14	2	14	12
1q21.1 duplication syndrome	14	4	28	10
7q11.23 deletion (Williams–Beuren syndrome, WBS)	14	3	21	11
17q12 deletion syndrome	6	3	50	3
17q12 duplication syndrome	6	2	33	4
22q11.2 distal deletion syndrome	5	2	40	3
22q11.2 distal duplication	5	3	60	2
16p12.1 deletion syndrome	10	3	30	7
Xp22.31 deletion (X‐linked Ichthyosis in male, XLI) in males	10	3	30	7
15q13.3 duplication	5	2	40	3
Syndromic pCNVs				
22q13.3 deletion (Phelan–Mcdermid syndrome, PMS)	9	2	22	7
22q13.3 duplication syndrome	2	1	50	1
17p13.3 deletion syndrome (Miller–Dieker lissencephaly syndrome, MDLS)	6	3	50	3
4p16.3 deletion (Wolf–Hirschhorn syndrome, WHS)	3	1	33	2
Total	266	100	38	166

## 4 DISCUSSION

**FIGURE 2:**
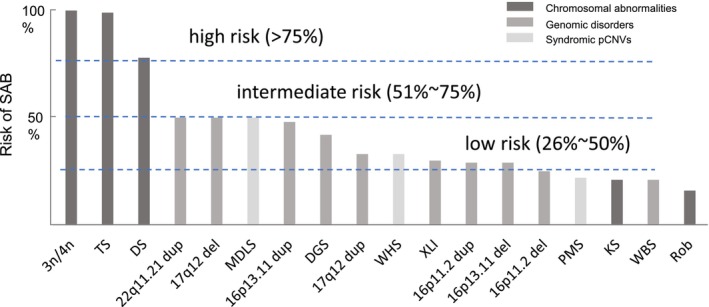
Risk of spontaneous abortions for major chromosomal abnormalities, genomic disorders, and syndromic pCNVs. Levels of low, intermediate, and high risk were indicated. 3n/4n, triploid/tetraploid; DS, Down syndrome; del, deletion; dup, duplication; KS, Klinefelter syndrome; MDLS, Miller–Dieker lissencephaly syndrome; PMS, Phelan–McDermid syndrome; Rob, Robertsonian translocation; TS, Turner syndrome; WBS, Williams–Beuren syndrome; WHS, Wolf–Hirschhorn syndrome; XLI, X‐linked ichthyosis.

Chromosomal abnormalities and pCNVs were detected in 49.9% and 2.5% of POC (Table 1, Figure 1) and 0.67% and 0.33% of pediatric patients, respectively (Chai et al., 2019). Higher incidence of cytogenomic abnormalities in POC reflected a natural selection against fetuses with these genomic imbalances. Several studies have noted the different incidences of genomic disorders and syndromic pCNVs in POC and newborns. The present study estimated an incidence of 1/1000 for DGS in POC, which is the same as that of a previous prenatal study (Grati et al., 2015) and falls between 1/500 from 2600 POC cases (Peng & Yuan, 2018) to 1/1500 from a large cohort of 22,451 POC samples (Maisenbacher et al., 2017). Higher incidence of DGS in POC than its newborn incidence of 1/4000–1/6000 suggests a high morbidity *in utero* and thus an increased risk of SAB. Similar incidence of WBS in POC and newborns was noted in Peng & Yuan, 2018, which explains the baseline risk of SAB for WBS. Therefore, the risk of SAB should be estimated for specific genomic disorders and syndromic pCNVs. It is well‐known that the risk of SAB for monosomy X (Turner syndrome, TS), unbalanced rearrangements, trisomy 21 (Down syndrome, DS), XXY (Klinefelter syndrome, KS), and Robertsonian translocations is 99%, 85%, 78%, 21%, and 16%, respectively (Nussbaum et al., 2016). Chromosomal abnormalities with a high risk of SAB (>75%) are frequently detected in POC from miscarriages, while some sex chromosome aneuploidies and balanced rearrangements showed a baseline risk of 15%–20% and more likely survive to term. Therefore, the levels of risk of SAB could be further defined as high risk (>75%), intermediate risk (51%–75%), low risk (26%–50%), and baseline risk (<25%). Of the 17 genomic disorders and four syndromic pCNVs summarized in Table 3, the risk of SAB for WBS (21%), PMS (22%), and 16p11.2 deletion (25%) was in the baseline level and therefore no significantly increased risk for SAB. The 22q11.2 distal duplication showed an intermediate risk of 60% for SAB, and all remaining genomic disorders and syndromic pCNVs had a low level of risk. The risk of SAB for representative chromosomal abnormalities, genomic disorders, and syndromic pCNVs is shown in Figure 2. Familial cases have been reported for genomic disorders and syndromic pCNVs. Approximately 7% of DGS inherited from a carrier parent with a deletion at 22q11.2 (McDonald‐McGinn et al., 2015), and about 7% of PMS had a parent carrying a balanced translocation involving 22q13 (Hao et al., 2022). The estimation on the levels of risk of SAB for these cytogenomic abnormalities provided evidence in prenatal diagnosis for laboratory interpretation and genetic counseling to evaluate the carrier status and reproductive risks for the parents.

Defining the risk of SAB for genomic disorders and syndromic pCNVs is essential to establish causal relation and elucidate genetic etiology of pregnancy losses in early human embryonic and fetal development. Genes in genomic disorders and pCNVs are functioned in dosage‐sensitive mechanisms of haploinsufficiency and/or triplosensitivity. ClinGen curated dosage‐sensitive genes are included in Table S1 for the listed genomic disorders and syndromic pCNVs. DGS is the most common genomic disorder with known haploinsufficient genes *HIRA* and *TBX1* at 22q11.2. The congenital malformations of heart defect, hypoplasia of the thymus, and hypoparathyroidism are often linked to the haploinsufficiency of the *TBX1* gene. However, recent studies revealed that genetic and epigenetic changes within and outside the 22q11.2 region can affect the levels of *TBX1* and dramatically sway the clinical phenotypes (Du et al., 2020). SAB for DGS could be the most severe phenotype from compound effects of heart failure and immunodeficiency. Bioinformatic approaches has been used to identify gene networks and functional pathways from pCNVs (Xu et al., 2014). An integrative gene discovery approach based on the genomic location, evolutionary conservation, human fetal or placental expression profile, and HiRVIS gene scores had been used on pCNVs from POC (Chen et al., 2017). This approach identified 244 putative candidate genes critical for human early embryonic development, neuronal development and differentiation, and transcriptional regulation of biological processes. The *CHRNA7* gene (OMIM*118511) at 15q13.3 is a known dosage‐sensitive gene among these candidate genes. The *CHRNA7* gene encodes nicotinic acetylcholine receptor alpha‐7 subunit, α7 nAChR, which plays roles on the calcium‐activated signal pathway and cholinergic anti‐inflammatory pathway (Di Lascio et al., 2022). A study on induced pluripotent stem cells derived from patients noted an unexpected down‐regulation of α7 nAChR‐associated calcium signal cascades in both deletion and duplication at 15q13.3 (Gillentine et al., 2017). Increased level of α7 nAChR correlated with excessive inflammation and synthesis of tumor necrosis factor and caused morbidity and mortality in diverse human diseases including endotoxemia, sepsis, rheumatoid arthritis, and inflammatory bowel disease (Di Lascio et al., 2022; Wang et al., 2003). Dysregulation of the two pathways modulated by *CHRNA7* may cause SAB. The genomic disorder with a mild phenotype in pediatric patients such as a deletion at 15q11.2 showed a 51% risk of SAB. This observation needed to be further confirmed from future studies. *In silico* analyses for the four coding genes of *NIPA1*, *NIPA2*, *CYFIP1*, and *TUBGCP5* in the 15q11.2 region suggested cardinal disease associations of *NIPA1*‐Spastic paraplegia 6, *NIPA2*‐PWS/AS, *CYFIP1*‐fragile X syndrome and autism, and *TUBGCP5*‐AS (Rafi & Butler, 2020). The functions of these four genes on early embryonic and fetal development should be explored. Variable expressivity resulted from dosage‐sensitivity genes and modifier genes related to genomic disorders, and syndromic pCNVs could also have an impact on the causality of SAB. A recent exome sequencing study on a cohort of POC indicated that genes with dominant effect on multisystem abnormalities, neurodevelopmental disorders, cardiac anomalies, skeletal dysplasia, metabolic disorders, and renal diseases could cause SAB (Zhao et al., 2021). Furthermore, maternal genetic polymorphisms and fetal microRNA polymorphisms associating with immunological responses, thrombophilia, abnormal placental function, and disturbance of metabolic regulation could have a modifying effect on the penetrance of SAB for chromosomal abnormalities and pCNVs (Salimi et al., 2022; Shi et al., 2017).An integrated approach should be applied to elucidate the dosage‐sensitive effect, dominant lethal conditions, genetic modifiers, and epigenetic changes related to pregnancy losses.

There were a few limitations in this study, and thus future studies to resolve these limitations should be considered. In the meta‐analysis, percentage of variation across studies that is due to heterogeneity rather than chance (*I* ^2^) is 99%, 98%, and 89% respectively (Figure 1), which indicates there is a very large difference between the seven studies (Higgins & Thompson, 2002). Finley's study contained the largest population with smallest bias. We decided to report the results from the common effect model instead of random effect from meta‐analysis in the main text (Table 1). We also included results of the random effect model in Figure 1 to show the heterogeneity of the seven studies and the possible variation of incidence in different populations. Chen's study included cases from China and USA, and results from CMA and next generation sequencing were included in Chen's and Li′s studies; these variations likely introduced an overestimation in the diagnostic yields; but the weight based on common effort was in the range of 4%–6.2%. It is necessary to collect more data and conduct meta‐analysis in different populations in the future. As shown in Table 2, the newborn incidence for genomic disorders and syndromic pCNVs from population genetic studies was available only for DGS, WBS, PMS, and XLI. The newborn incidences for other genomic disorders and syndromic pCNVs estimated from the diagnostic outcome of a large pediatric cases series (Girirajan et al., 2012) were consistent with that from a previous estimation on two large case series (Wei et al., 2013). However, the diagnostic efficacy for genomic disorders in the current pediatric setting was estimated about 46% with variations from 18% for 1q21.1 deletion to 92% for DGS (Chai et al., 2019). The yield of pCNVs was positively correlated with phenotypic complexity and age of onset (Yuan et al., 2021). Therefore, the newborn incidences from this approach were an under‐estimation. An estimation of the prevalence of genomic disorders using CMA data in a linear regression model showed population prevalence of 1/3000 to 1/6900 for 1q21,1 microdeletion/microduplication syndromes, 15q13.3 deletion syndrome, 16p11.2 microdeletion syndrome, and 16p12.2 microduplication syndrome. This approach represented an underestimation of population prevalence and cannot directly convert to newborn incidences (Gillentine et al., 2018). Furthermore, different newborn incidences of DGS from 1/4000 to 1/6500 were noted in different ethnic groups (Botto et al., 2003; Goodship et al., 1998). A comparison of pCNVs between Chinese and Western patient cohorts showed different diagnostic yields and clinical features in 15 known genomic disorders (Yuan et al., 2021). Gender bias with female predilection 2:1 for WHS and JS should also be taken into consideration (Ji et al., 2010; Maas et al., 2008). The genomic structure of locus‐specific low copy repeats from a common ancestor could affect the newborn incidences in certain ethnic groups. For example, the high frequency of an inversion at 17q21 predisposed a high newborn incidence of 17q21 microdeletion in Southern Europe and Southwest Asia populations (Donnelly et al., 2010). Therefore, a large‐scale population genetic study with considerations on diagnostic efficacy and different ethnic groups is needed to give a more reliable evaluation of newborn incidences for genomic disorders and syndromic pCNVs. Nevertheless, the risk of SAB for DGS, WBS, XLI, and WHS was based on newborn incidences from population genetic studies and the risk of SAB for other pCNVs could be slightly an overestimation due to the underestimation of newborn incidence from diagnostic yields.

In conclusion, this study summarized the genomic disorders and syndromic pCNVs detected in POC from seven large case series and estimated the incidence of these pCNVs in pregnancy losses. The risk of SAB for major genomic disorders and syndromic pCNVs was estimated through a comparison of their incidences in POC and pediatric patients. Further classification on levels of risk of SAB was provided for major chromosomal abnormalities, genomic disorders, and syndromic pCNVs. These results could provide evidence‐based interpretation of detected genomic disorders and syndromic pCNVs in prenatal diagnosis and insight into genetic and pathologic correlations of pCNVs with pregnancy losses.

## AUTHOR CONTRIBUTIONS

Qinghua Zhao, Hongyan Chai, and Jiadi Wen performed the literature search, data retrieval, variant re‐evaluation, and statistical analysis, Gang Peng and Hongyu Zhao performed meta‐analysis, checked MOOSE guidelines, and statistical interpretation. Peining Li designed the study, interpreted the results, and drafted the manuscript. Hugh Taylor and Yong‐Hui Jiang provided suggestions to the study design and critical revisions on the content. All authors discussed and reviewed the manuscript and approved its final version.

## FUNDING INFORMATION

This study was partly supported by NIH/NICHD grant 1 R01 HD105267‐01 (Large scale genome sequencing and integrative analyses to define genomic predictors of recurrent pregnancy loss) to Hugh Taylor and Yong‐Hui Jiang.

## CONFLICT OF INTEREST STATEMENT

The authors declare no conflicts of interests.

## Supporting information


Table S1.

## Data Availability

The original data extracted from large case series of CMA on POC in this study are openly available in PubMed (https://pubmed.ncbi.nlm.nih.gov/), and the summarized data are presented in the main text and supplementary material of this article.
